# A Textbook of Legal Medicine and Toxicology

**Published:** 1904-12

**Authors:** 


### BOOK REVIEWS.

A TEXTBOOK OF LEGAL MEDICINE AND TOXICOLOGY. Edited by FREDERICK PETERSON, M.D., Chief of Clinic, Nervous Department of the College of Physicians and Surgeons, New York; and Walter S. Haines, M.D., Professor of Chemistry, Pharmacy, and Toxicology, Rush Medical College, in affiliation with the University of Chicago. Two imperial octavo volumes of about 750 pages each, fully illustrated. Philadelphia, New York, London: W. B. Saunders & Company. 1903. (Per volume: cloth, $5.00 net; sheep or half morocco, $6.00 net.)

The second volume of this work maintains the high standard which characterised volume one. The authors of the several articles have been wisely made responsible for their statements by the editors. This is of importance in medico-legal works, as the author and not the editor is the one who is quoted. In the article on legitimacy, by J. F. Darling and Goldwater, we find many interesting facts which will make an expert cautious when delivering an opinion as to paternity. Thus Hirst is quoted as having a record of a boy of 13, who became a father, and two authentic instances of paternity at 82, and over 100 years respectively. The period of maternity is stated to be between the ages of 8 and 55. In this connection it must be remembered that Fordyce Barker declared "that the laws of physiology, the experience of mankind and the decisions of the courts of law, justify a medical man in declaring that a woman over 55 years of age is past the period of childbearing."

As the subject of prolonged pregnancy receives careful attention, the tables of Merriman and Montgomery are quoted, showing that pregnancy has been prolonged to 302 days.

Impotence and sterility, rape and unnatural sexual offences are interestingly and thoroughly discussed by Charles Gilbert Chaddock. The article on malpractice is of especial importance to all physicians. The contributor, Marshall D. Ewell, a professor of medical jurisprudence in Chicago, has laid down with rare skill the various rules held in different cases in civil and criminal malpractice.

Carlos F. MacDonald has arranged alphabetically the laws relating to the insane. This is a digest of the statutes of all states and territories and of the District of Columbia. This article completes Part I.

Part II. is devoted to toxicology and all other divisions of legal medicine in which laboratory investigation is an essential feature.

Poisons are divided by the editor, W. S. Haines, into inorganic, alkaloidal, nonalkaloidal, organic, gaseous and, lastly, food poisons. The diagnosis of poisoning, the treatment of the same, and the method of conducting a postmortem in a poison case are all carefully described. The chemical analysis by different methods and their relative values are clearly set forth. The postmortem changes of tissues are shown by beautifully colored plates.

The chemical articles are contributed by Haines, Holland, Prescott, Reid Hunt, Walter Jones, Doremus, and Harrington.

Victor Vaughan in the article on ptomains calls special attention to the difficulties of separating vegetable alkaloids from putrefactive bodies. Among all the schemes presented for this purpose he says, "that devised by Kippenberger is the only one worthy of mention." Vaughan says further of this method from his own experience, that it does not wholly solve the difficulties of separating morphine from putrefactive bodies that respond to some of the morphine tests.

The examination of blood and blood stains by Edward S. Wood, professor of chemistry in the Harvard Medical School, ends with the following conclusions: (1) "The majority of hematologists agree that discrimination can often be made in the case of dried blood stains between human blood and that of domestic animals; (2) the serum test is the most delicate one thus far discovered for differentiating between the different kinds of blood and has been shown to be applicable to both fresh and decomposed blood and to dried blood stains."

The two volumes are ably edited and cannot fail to be of great value to all physicians who have any medico-legal work. We cordially recommend it as one of the most useful textbooks on legal medicine. The work of the publishers leaves nothing to be desired. J. W. P.

